# Polymerase II Promoter Strength Determines Efficacy of microRNA Adapted shRNAs

**DOI:** 10.1371/journal.pone.0026213

**Published:** 2011-10-21

**Authors:** Robert Jan Lebbink, Maggie Lowe, Theresa Chan, Htet Khine, Xiaoyin Wang, Michael T. McManus

**Affiliations:** Department of Microbiology and Immunology, Diabetes Center, University of California San Francisco, San Francisco, California, United States of America; Beckman Research Institute of the City of Hope, United States of America

## Abstract

Since the discovery of RNAi and microRNAs more than 10 years ago, much research has focused on the development of systems that usurp microRNA pathways to downregulate gene expression in mammalian cells. One of these systems makes use of endogenous microRNA pri-cursors that are expressed from polymerase II promoters where the mature microRNA sequence is replaced by gene specific duplexes that guide RNAi (shRNA-miRs). Although shRNA-miRs are effective in directing target mRNA knockdown and hence reducing protein expression in many cell types, variability of RNAi efficacy in cell lines has been an issue. Here we show that the choice of the polymerase II promoter used to drive shRNA expression is of critical importance to allow effective mRNA target knockdown. We tested the abundance of shRNA-miRs expressed from five different polymerase II promoters in 6 human cell lines and measured their ability to drive target knockdown. We observed a clear positive correlation between promoter strength, siRNA expression levels, and protein target knockdown. Differences in RNAi from the shRNA-miRs expressed from the various promoters were particularly pronounced in immune cells. Our findings have direct implications for the design of shRNA-directed RNAi experiments and the preferred RNAi system to use for each cell type.

## Introduction

RNA interference (RNAi) is a process of double-stranded RNA-dependent post-transcriptional gene silencing. It has become the most powerful and widely used strategy for genetic analysis based on the highly specific and efficient silencing of target genes [1], [2], [3]. Upon cell entry, double-stranded RNA is cleaved by the RNAse III enzyme Dicer into double-stranded small interfering RNAs (siRNAs) of ∼21–23 nt in length with a two-base 3′ overhang [4], [5]. These short-interfering RNAs (siRNAs) are subsequently incorporated into the RNA-induced silencing complex (RISC), which uses one strand of the siRNA as a guide to target the complementary mRNA for degradation by RNA cleavage directed by Ago2 [6]. In this manner, RNAi allows for the sequence-specific degradation of mRNAs expressed in the cell, thereby lowering the expression level of the encoded proteins (protein knockdown).

The discovery of RNAi has led to the development of methods that usurp the RNAi pathway to specifically degrade specific mRNA molecules thereby allowing loss-of-function phenotype studies in mammalian cells. Of these, commercial synthetic siRNAs are most widely used as means to target complementary cellular mRNAs[7]. More recently much attention has been given to the development of genetically encoded siRNAs by expressing so-called short hairpin RNAs (shRNAs) in a cell, consisting of a sequence of ∼21–25 nt, a short loop region, and the reverse complement of the 21- to 25-nt region driven by a polymerase (pol) III promoter such as U6 or H1 [3], [8], [9], [10], [11]. When transcribed in vivo, this short transcript forms a hairpin structure, which is subsequently converted by Dicer into short RNAs that are recognized by the RNA-induced silencing complex and induce mRNAs cleavage.

The shRNA stem-loop is structurally related to microRNA (miRNA) precursor stem-loop RNAs. miRNA precursors encode a highly conserved class of endogenous 21 to 23-nucleotide-long microRNAs. These small RNAs were originally described in worms and display less complete sequence complementarity to their targets as compared to shRNAs [12], [13], but also act by inducing gene silencing [12], [13], [14], [15], [16]. miRNAs are transcribed as part of long primary transcripts (pri-miRNAs); the pri-miRNAs are cleaved by the nuclear Drosha-DGCR8 microprocessor complex to produce approximately 70 nt stem-loop structures of precursor miRNAs (pre-miRNAs). The pre-miRNAs are subsequently transported from the nucleus to the cytoplasm by Exportin-5, where they are subjected to cleavage by the RNase III enzyme Dicer to yield a 20–23 nt double-stranded RNA siRNA of which the mature miRNA strand is loaded into RISC, and guides mRNAs target cleavage or more often inhibition of protein synthesis (reviewed in Ku and McManus [1]).

Whereas polymerase III expressed shRNAs are modeled after precursor miRNAs, other systems are modeled after primary miRNA transcripts in which the shRNA is flanked by genomic miRNA sequences that are naturally present in miRNA genes [17], [18], [19], [20], [21], [22], [23]. These microRNA-adapted shRNAs (shRNA-miRs) are expressed from polymerase II promoters and feed into the miRNA biosynthesis pathway upstream the microprocessor cleavage step. Since these shRNA-miRs usurp the entire miRNA processing machinery, they are considered to be a more ‘natural’ system to induce RNAi; additionally several reports have suggested that these shRNAs are more potent in driving target knockdown as polymerase III expressed shRNAs [19], [20], [21], [24], [25]. These systems have been mass-produced in lentiviral vectors since lentivirus offers shRNA introduction into a wide variety of cell types. In addition, many– if not most lentiviral shRNA constructs use the CMV pol II promoter to drive shRNA expression, given the promoter's activity in a wide variety of cell types.

We observed that shRNA-miRs expressed from commonly used polymerase II promoters are often less effective in driving target-gene knockdown as compared to ‘conventional’ shRNAs expressed from the polymerase III U6 promoter. Our studies indicate that the potency of knockdown using shRNA-miRs is largely determined by the relative promoter strength of the polymerase II promoter that drives expression of the shRNA, and that large variations in target knockdown occur due to the differential potency of such promoters in different cell types. These data support a model where small RNA abundance is a limiting factor for the activity of shRNAs and perhaps microRNAs. To our knowledge this has not been formally tested in the context of shRNAs. Importantly, our findings have direct implications for the design of shRNA-directed RNAi experiments and the choice which RNAi system to use for which cell type.

## Results

### anti-EGFP shRNAs expressed from a miRNA backbone are less efficient in target-knockdown in Jurkat T cells as compared to 293T cells

**Figure 1:**
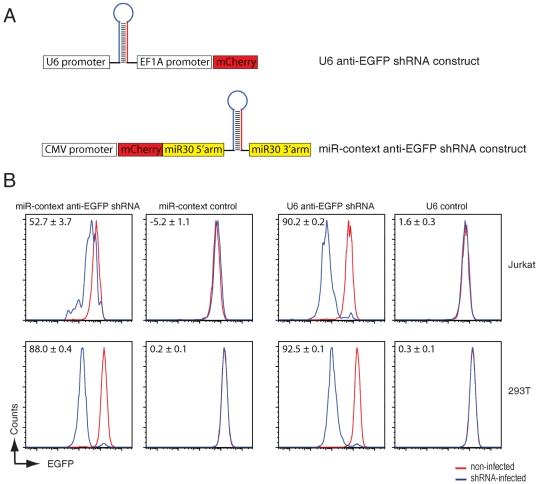
anti-EGFP shRNAs expressed from a miRNA backbone are less efficient in target-knockdown in Jurkat T cells than in 293T cells. A) schematic representation of the miR-context anti-EGFP shRNA construct cloned in the 3′UTR of mCherry which is expressed under the control of the polymerase II CMV promoter (lower panel) and the control anti-EGFP shRNA expressed from a mouse polymerase III U6 promoter (top panel). B) EGFP-expressing Jurkat T cells (top panels) or 293T cells (lower panels) were infected at an MOI of <0.2 with anti-EGFP shRNAs expressed from a mouse U6 promoter (U6 anti-EGFP shRNA) or from a miR-30 backbone expressed from a CMV promoter (miR-context anti-EGFP shRNA) or the relevant control viruses that lack shRNA inserts. Cells were allowed to grow for 8 days and the expression of EGFP (to monitor knockdown) and mCherry (as marker for infected cells) were assessed by flow cytometry. The indicated percentage of EGFP knockdown is calculated by ((Geo-mean of uninfected cells minus Geo-mean of infected cells)/Geo-mean of uninfected cells)*100. The presented data is a representative experiment of 2 experiments performed in triplicate.

**Figure 2:**
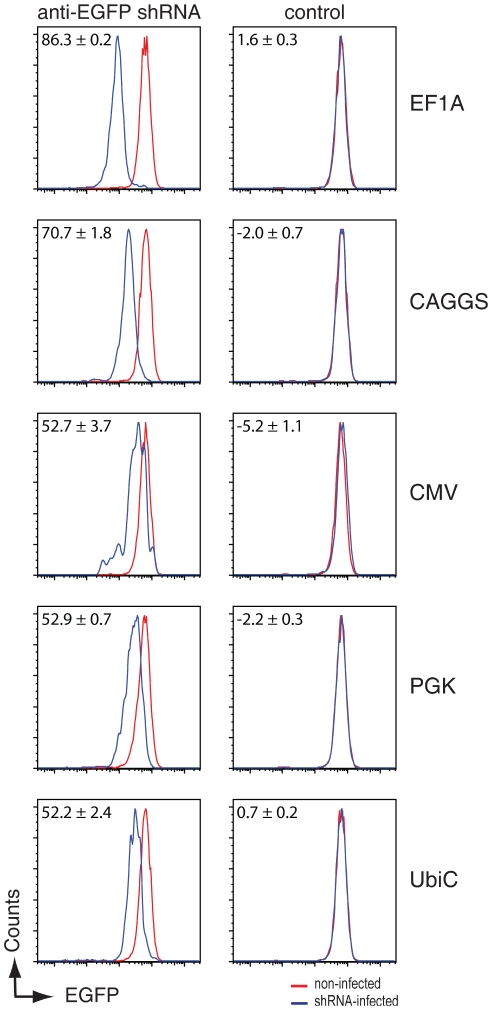
shRNA-miR-directed target knockdown is highly dependent on the polymerase II promoter used. EGFP-expressing Jurkat T cells were infected at an MOI of <0.2 with anti-EGFP shRNA-miRs (left panels) or controls (no shRNA insert, right panels) driven by various indicated polymerase-II promoter. Cells were allowed to grow for 8 days and the expression EGFP (to monitor knockdown) and mCherry (as marker for infected cells) were assessed by flow cytometry. The indicated percentage of EGFP knockdown is calculated by ((Geo-mean of uninfected cells minus Geo-mean of infected cells)/Geo-mean of uninfected cells)*100. The presented data is a representative experiment of 2 experiments performed in triplicate.

In the process of generating stable EGFP knockdown cell lines in human Jurkat T cells by using an anti-EGFP shRNA expressed from CMV-driven miR30-backbone expression vectors (shRNA-miRs, see Figure 1A for design of the constructs), we noted a much reduced target knockdown (52.7%) as compared to identical shRNAs expressed from the mouse polymerase III U6 promoter (90.2%, see Figure 1B, top panels). These same lentiviral shRNA vectors were equally potent in driving EGFP knockdown in human 293T cells (88.0% and 92.5% target knockdown for shRNA-miR and U6-driven shRNAs respectively, see Figure 1B lower panels), suggesting that shRNA-miRs might be less potent in Jurkat T cells as in 293T cells. We set out to explore the nature of this discrepancy, and assessed whether the potency of the polymerase II promoter used to express the miR30-backbone anti-EGFP shRNAs could impact the shRNA-directed silencing in Jurkat T cells. To this end, we cloned five different polymerase II promoters upstream the miR-30 anti-EGFP shRNA cassette that is located in the 3′UTR of the fluorescent protein mCherry to mark cells that have been infected with the lentiviral construct. We selected the ubiquitin C (UbiC) promoter, the phosphoglycerate kinase (PGK) promoter, the cytomegalovirus (CMV) promoter, a beta-actin promoter with an upstream CMV IV enhancer (CAGGS), and the elongation factor 1 alpha promoter (EF1A) since these promoters are among the most commonly used polymerase II promoters to drive mRNA expression. We analyzed the potency of these promoters in driving anti-EGFP shRNA expression by measuring EGFP protein knockdown in EGFP-expressing cells via flow cytometry (See Figure 2). shRNAs expressed from these promoters displayed various potency in target-knockdown in Jurkat cells. The ubiquitin C (UbiC) promoter, the phosphoglycerate kinase (PGK) promoter, and the cytomegalovirus (CMV) promoter were equally potent in driving EGFP-target knockdown (52.2%, 52.9%, and 52.7% respectively), whereas the beta-actin promoter with an upstream CMV IV enhancer (CAGGS, 70.7%), and the elongation factor 1 alpha promoter (EF1A, 86.3%) were much more effective (See Figure 2). These data reveal a significant impact of the choice of polymerase II promoter used in shRNA-directed target knockdown in human Jurkat T cells.

### shRNA-miRs-directed target knockdown is dependent on cell type and polymerase II used

**Figure 3:**
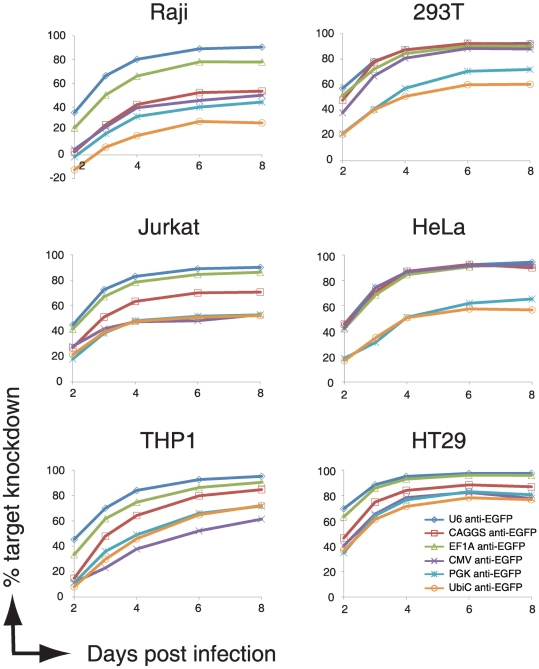
Efficacy of shRNA-miRs-guided target knockdown in various cell lines is dependent on the type of polymerase II promoter used. EGFP-expressing human immune cell types (Raji B cells, Jurkat T cells, and THP-1 monocytic cells) and adherent cell lines (293T, HeLa, and HT29 cells) were infected at an MOI of <0.2 with anti-EGFP shRNAs expressed from a miR-30 backbone driven by various indicated polymerase-II promoter. Cells were allowed to grow for the indicated number of days and the expression of EGFP (to monitor knockdown) and mCherry (as marker for infected cells) was assessed by flow cytometry. The presented percentage of EGFP knockdown is calculated by ((Geo-mean of uninfected cells minus Geo-mean of infected cells)/Geo-mean of uninfected cells)*100. The presented data is a representative experiment of 2 experiments performed in triplicate. For all data points, the standard deviation was below 5%.

RNAi is often performed in cells that are relatively easy to grow and transfect with commercially available lipids. We asked whether any of the broadly used cell lines would display differing RNAi effectiveness using our panel of promoters expressing the same shRNA. To address this question we chose to monitor three commonly used adherent cell lines (293T embryonic kidney cells, HeLa cervical carcinoma cells, and HT29 adenocarcinoma cells) and three commonly used non-adherent immune cell lines (Jurkat T cell lymphoblast cells, Raji Burkitt lymphoma B cells, and THP-1 acute monocytic leukemia cells). Stable EGFP-expressing cells were generated for all six lines, and infected at a low MOI with the various viruses. EGFP expression was then monitored at 2, 3, 4, 6, and 8 days post infection by flow cytometry (See Figure 3). In all cases, the EF1A-anti-EGFP construct was most efficient in driving EGFP knockdown as compared to the other four polymerase II-promoters. This observation was most prominent in the non-adherent immune cell types (Raji, Jurkat, and THP1 cells, See Figure 3 left panels). In general EGFP-knockdown was more variable using the different polymerase-II promoters in immune cells as compared to the adherent lines. The EF1A, CAGGS and CMV promoters were equally potent in 293T and HeLa cells, whereas the PGK and UbiC promoters displayed much-reduced EGFP-target knockdown in these lines. Optimal EGFP-knockdown was observed 6 to 8 days post infection for all promoters in all lines. In Table 1 we present a summarizing overview of the RNAi efficiencies of the tested promoters in the different cell lines.

**Table 1:** Overview of the efficacy of shRNA-miRs-guided target knockdown in various human cell lines.

	Raji	Jurkat	THP1	293T	HeLa	HT29
**U6**	90.5±0.3	90.2±0.2	95.2±0.1	92.5±0.1	92.3±0.2	97.4±0.1
**EF1A**	80.0±2.2	86.3±0.2	90.5±0.2	90.2±0.1	90.7±0.1	95.8±0.1
**CAGGS**	53.4±0.9	70.7±1.8	84.7±0.7	91.8±0.1	89.9±4.3	86.8±0.8
**CMV**	50.0±1.6	52.7±3.7	61.5±3.0	88.0±0.4	91.9±0.2	77.7±0.3
**PGK**	44.3±1.2	52.9±0.7	71.8±0.8	71.9±3.3	65.3±3.8	80.6±0.3
**UbiC**	26.8±1.0	52.2±2.4	72.2±2.4	60.2±0.4	56.7±4.9	76.7±0.7

EGFP-expressing human cell lines (see top row) were infected at an MOI of <0.2 with anti-EGFP shRNAs expressed from a miR-30 backbone driven by various indicated polymerase-II promoters (left column). Cells were allowed to grow for 8 days and the percentage of EGFP target knockdown was assessed by flow cytometry (see Figure 3 for details). The presented data is a representative experiment of 2 experiments performed in triplicate.

**Figure 4:**
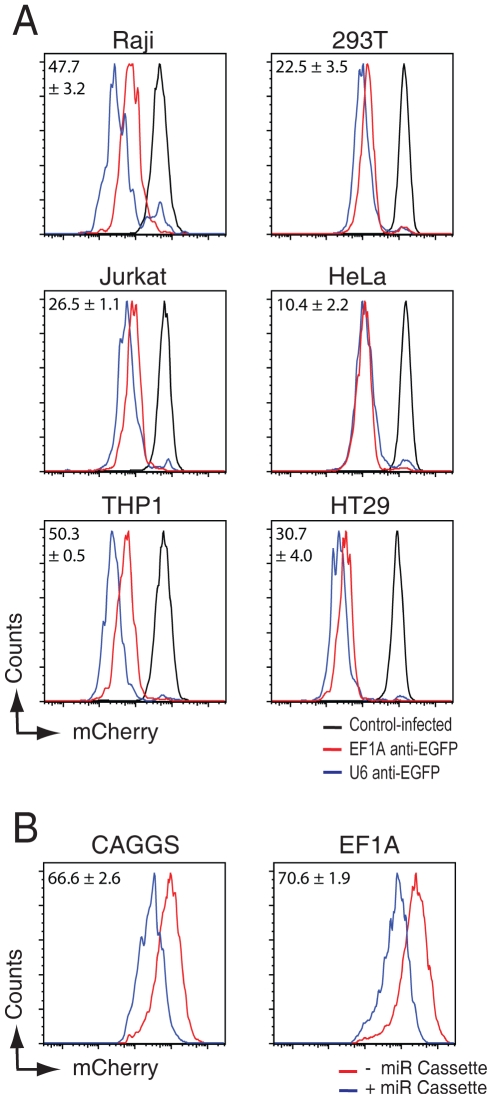
anti-EGFP shRNAs expressed from a mouse U6 promoter is more potent as compared to anti-EGFP shRNA-miRs expressed from an EF1A-promoter. A) EGFP-expressing human immune cell types (Raji B, Jurkat T, and THP-1 monocytic cells) and adherent cell lines (293T, HeLa, and HT29 cells) were infected at an MOI of <0.2 with anti-EGFP shRNAs expressed from a mouse U6 promoter (blue histograms) or with anti-EGFP shRNA-miRs expressed from the human EF1A promoter (red histograms). Eight days post infection, infected cells were monitored for EGFP expression by flow cytometry. The presented percentage of EGFP knockdown of the U6-driven shRNAs relative to the EF1A-driven shRNAs is calculated by ((Geo-mean of EF1A infected cells minus Geo-mean of U6 infected cells)/Geo-mean of EF1A infected cells)*100. B) mCherry expression is reduced upon cloning of an shRNA-containing miR-30 cassette in the 3′UTR of the fluorescent protein. Two examples are shown where either the CAGGS or EF1A promoter drives expression of mCherry with/without a functional miR cassette in it's 3′UTR. The percentage of reduced relative mCherry expression caused by the miR-cassette containing UTR is indicated. The presented data are representative experiments of 2 experiments performed in triplicate.

Although some groups reported that miR-backbone expressed shRNAs are more potent as polymerase III U6-promoter expressed shRNAs [19], [21], [24], [25], we did not observe this result. In contrast, the U6 promoter expressed shRNAs were more potent in directing target knockdown as compared to the most effective EF1A promoter in all 6 cell lines (See Figure 3 and Figure 4A). The U6 promoter caused an additional target knockdown of 22.5%, 10.4%, and 30.7% in 293T, HeLas, and HT29 cells as compared to the EF1A promoter (see Figure 4A). This enhanced target knockdown was even more pronounced in the immune lines Raji, Jurkat and THP1 in which an additional 47.7%, 26.5%, and 50.3% of target knockdown was achieved over the EF1A promoter (Figure 4A). We conclude that shRNA-miRs efficacy is largely dependent on the polymerase II used to drive expression of the shRNA, and that this differential target knockdown is dependent on the cell type used.

### Promoter strength determines the potency of miRNA backbone-expressed shRNAs

We assessed whether EGFP-target knockdown in the tested cell lines correlates with the relative polymerase II promoter strength in those lines. To determine the relative strength of the promoter, we measured the mCherry transgene fluorescence as a proxy for promoter activity. However, when shRNAs were present within the 3′UTR of the mCherry transgene, we noted a clear reduced expression (up to 70.6%) of the mCherry gene cloned immediately upstream of the shRNA-miR (see Figure 4b). This reduced expression of the reporter is likely due to competition between mRNA export and microprocessor processing of the anti-EGFP shRNA out of the mCherry 3′UTR, thereby destabilizing the mCherry transcript. Since this processing could be impacted by the number of mRNA copies present in the cell, we thus chose to correlate the relative mCherry expression with viruses not expressing the miR-30 cassette to the percentage of target knockdown in cells expressing the shRNAs.

**Figure 5:**
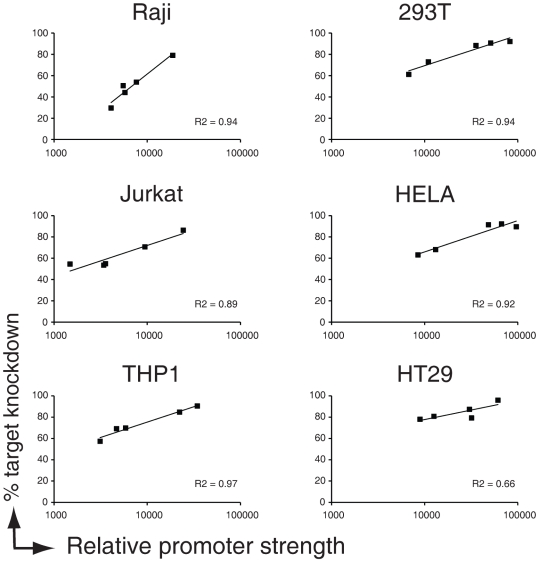
Promoter strength determines potency of shRNA-miRs. EGFP-expressing human immune cell types (Raji B, Jurkat T, and THP-1 monocytic cells) and adherent cell lines (293T, HeLa, and HT29 cells) were infected at an MOI of <0.2 with anti-EGFP shRNAs (left panels) expressed from a miR-30 backbone driven by various polymerase-II promoter. Cells were allowed to grow for 8 days and the expression of EGFP (to monitor knockdown) was assessed by flow cytometry. Relative promoter strength of the polymerase II promoters (mCherry Geo Mean) was measured from control vectors that lacked the miR30-backbone, since processing of shRNAs out of the miR30 backbone destabilized the transcript. Relative promoter strength is plotted against% EGFP knockdown in the indicated cellines. Trendlines and R^2^ values of fitted curves are indicated.

To this end, we measured the relative strength of the promoters by assessing the mCherry fluorescence of infected cells 8 days post infection and plotted this to the percentage of target knockdown at this same time point (see Figure 5). For all lines, there was a clear correlation between promoter strength (as determined by mCherry expression) and EGFP-knockdown by the miR30-backbone anti-EGFP shRNA, suggesting that the strength of the promoter is a major determinant for shRNA-miR potency.

### ‘Mature’ anti-EGFP siRNA expression levels correlate with target-knockdown

**Figure 6:**
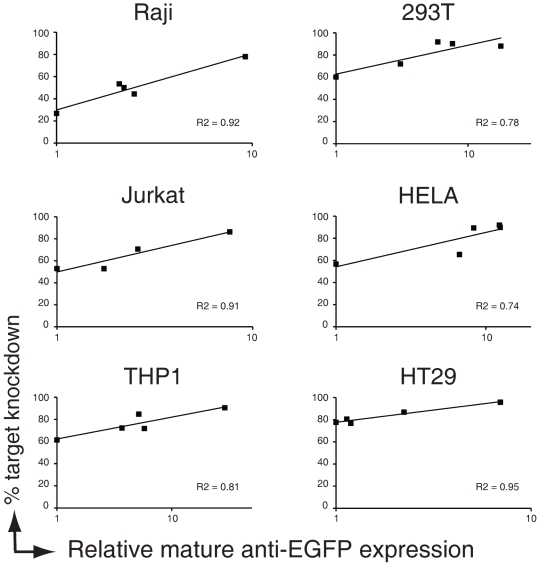
Relative quantity of anti-EGFP siRNAs correlates with target-knockdown. EGFP-expressing human immune cell types (Raji B, Jurkat T, and THP-1 monocytic cells) and adherent cell lines (293T, HeLa, and HT29 cells) were infected at an MOI of <0.2 with anti-EGFP shRNAs (left panels) expressed from a miR-30 backbone driven by various polymerase-II promoters. Cells were allowed to grow for 8 days and the expression of EGFP (to monitor knockdown) was assessed by flow cytometry. Total RNA was isolated and relative mature anti-EGFP siRNA expression was determined by TaqMan analysis. The lowest quantity of mature anti-EGFP within each cell line was set to 1. Relative mature anti-EGFP siRNA expression is plotted against% EGFP knockdown in the indicated cellines. Trendlines and R^2^ values of fitted curves are indicated.

If promoter strength is a critical variable for induction of potent RNAi, the amount of small RNAs should correlate with the activity of the promoter. To formally test this we quantitated the relative expression levels of the fully processed anti-EGFP siRNA antisense strand in the cells expressing the various promoter constructs. To this end, we designed TaqMan qPCR primers and probes specific for the ‘mature’ anti-EGFP siRNA based on the validated TaqMan approach for mature miRNAs [28]. The qPCR was specific for the mature anti-EGFP siRNA strand (data not shown) and efficiently amplified this species in our cell lines expressing anti-EGFP shRNAs driven by either the U6 promoter or the various polymerase II promoters. We sorted cells expressing single copies of the various anti-EGFP expressing shRNAs vectors by FACS, and assessed the relative expression level of mature anti-EGFP siRNAs herein and plotted this to the percentage of protein target knockdown at this same timepoint (see Figure 6). Again, for all lines, there was a clear correlation between promoter strength (as determined by mature anti-EGFP siRNA expression) and EGFP-knockdown by the anti-EGFP shRNA. We conclude that a more potent polymerase II promoter yields higher quantities of anti-target siRNAs, resulting in higher protein target knockdown. Of note, since we quantitated the EGFP target knockdown by measuring the amount of fluorescent protein in the cells and not by assessing the EGFP mRNA levels, we cannot account for the impact of differences in EGFP stability on RNAi in these different cell types.

## Discussion

Since the discovery of RNAi and microRNAs more than a decade ago, an incredible amount of research has been focused on the development of systems that usurp miRNA biology as a tool to downregulate protein expression in mammalian cells [1], [2], [3]. Recent progress has been made with the development of shRNA-miRs, in which single or even multiple endogenous miRNA pri-cursors are expressed from polymerase II promoters and the mature microRNA sequence is replaced by gene specific duplexes that guide RNAi [17], [18], [19], [20], [21], [22], [23]. Although these tools proved very effective in directing target mRNA knockdown and hence reduced protein expression in many cell types, we observed that the choice of the polymerase II promoter used to drive shRNA expression is of critical importance to allow effective mRNA target knockdown. We observed a clear positive correlation between promoter strength and protein target knockdown.

Researchers are often not aware that promoter choice directly impacts target knockdown efficiency and hence could determine the success of an experiment. We especially caution researchers that utilize commercial shRNA-miR expression vectors in immune cells, since these often make use of CMV promoters to drive expression of gene-specific shRNA-miRs. Although the CMV promoter causes very efficient expression in many cancer cell lines of fibroblast and epithelial origin, this promoter is much less active in lymphohematopoietic cells such as T, B and monocytic cells as we and others [29], [30] have shown. This often-underappreciated fact not only impacts transgene expression of protein coding genes in immune cells, but also negatively impacts the expression levels of shRNA-miRs thereby lowering the silencing capacity of these RNAi molecules. Since we show a clear positive correlation between promoter strength, the level of ‘mature’ siRNA expression, and protein target knockdown, we recommend using promoters that have maximum promoter strength in the target cell of interest to allow optimum protein knockdown using shRNA-miRs in mammalian cells.

A number of reports have indicated that expression of shRNAs via polymerase III promoters can cause toxicity *in vivo* in shRNA-expressing cells [31], [32], [33], whereas expression of shRNAs from a polymerase II promoter attenuates this effect [32], [34]. These shRNA-induced toxicities are believed to be mediated via saturation of endogenous RNAi export machinery [31], [34] or via off-target silencing of unintended mRNAs through buildup of antisense RNAs [32]. We have not observed delayed growth of cells expressing shRNAs from the polymerase III U6 promoter in our experiments (data not shown), and hence have no evidence of toxic effects by shRNA expressed via this system. Nevertheless, we cannot exclude that overexpressing shRNAs through very potent polymerase II or III promoters could induce toxicity in other *in vitro* or *in vivo* settings via this saturation effect. Although this potential toxic effect may or may not be a concern in experiments with shRNAs in cell lines, caution is necessary when studying the *in vivo* roles of genes or when designing shRNAs for therapeutic purposes.

We and others [35] have noted that cloning of shRNA-miRs downstream of a reporter gene causes reduced protein expression of the upstream gene. This observation is likely caused by processing of the anti-EGFP shRNA out of the reporters 3′UTR by the microprocessor, thereby destabilizing the mRNA transcript. This observation proved especially problematic in cells where the polymerase II promoter drives naturally low expression of the reporter gene, since shRNA clipping reduced reporter expression to (almost) undetectable levels (data not shown). To allow optimal detection of reporter expression of shRNA-miR containing expression vectors, the reporter gene(s) should be expressed from a different polymerase II promoter.

Although U6 driven shRNA expression is optimal for efficient target knockdown in a broad range of cell lines, shRNA-miRs expressed from polymerase II promoters allow for cell or tissue specific silencing of target mRNAs. This feature may be particularly useful to study the function of genes in a specific cell type in stable knockdown mice. However, in order to obtain good target knockdown, tissue specific expression of shRNA-miRs alone is not adequate, as the promoter that drives expression of the shRNA-miR should also be of sufficient strength in order to allow efficient target knockdown.

## Materials and Methods

### Cells and cDNA

Cell lines were obtained from the American Type Culture Collection (Manassas, VA) and cultured using standard techniques. Human cell lines that were used in this study are: 293T embryonic kidney cells, HeLa cervical carcinoma cells, HT29 adenocarcinoma cells, Jurkat T cell lymphoblast cells, Raji Burkitt lymphoma B cells, and THP-1 acute monocytic leukemia cells.

The pSicoR-EGFP lentiviral vector [8] was used as backbone vector to express EGFP and a puromycin resistence gene under control of the EF1A promoter. These proteins were fused by the viral T2A sequence [26] to allow expression of two separate proteins from the same mRNA transcript.

An anti-EGFP shRNA sequence (5′-GCAAGCTGACCCTGAAGTTC**TTCAAGAGA**GAACTTCAGGGTCAGCTTGCTTTTTT-3′) was cloned downstream of the polymerase III U6 promoter in pSicoR [8]; this vector also encodes mCherry under the control of the EF1A promoter. The loop of the shRNA is indicated in bold and the polymerase III terminator is underlined. A similar anti-EGFP shRNA sequence with the miR30 loop (bold) was cloned between miR30 pricursor arms (underlined and italic) downstream of mCherry in the same vector (*AAGAAGGTATATTGCTGTTGACAGTGAGCG* TCAAGCTGACCCTGAAGTTCAT**TAGTGAAGCCACAGATGTA**ATGAACTTCAGGGTCAGCTTGC*TGCCTACTGCCTCGGACTTCAAGGGG* ), the U6 promoter unit was removed.

Various mammalian and viral polymerase II promoters (CMV, PGK, UbiC, CAGGS, EF1A) were cloned upstream of mCherry in the miR30-anti-EGFP vectors and control (mCherry alone) vectors. The CMV and PGK promoters were amplified from pSicoR and pSicoR PGK respectively [8], the UbiC promoter was obtained from pDSL_hpUGIH (ATCC), the CAGGS promoter was amplified from pCAGGS (BCCM), and the EF1A promoter was amplified from the pEF6 vector (Invitrogen). All constructs were sequence verified by conventional DNA sequencing technologies.

### Virus Generation and generation of stable transfectants

Lentiviruses were generated essentially as described [27]. Briefly, 4 µg of lentiviral vector and 1.33 µg of each packaging vector (pMD2G-VSVg, pRSV-REV, and pMDL/RRE) were cotransfected in 293T cells by using the FuGENE 6 reagent (Roche Diagnostics). Supernatants were collected 48 h after transfection, filtered through a 0.25-µm filter, and used directly to infect target cells. For infections of suspension cells (Raji, Jurkat, THP-1), ∼5×10^5^ cells were spinfected for 1.5 hours at 1000 g at 33°C in 24 well-plates in the presence or ∼1×10^5^ lentiviral particles in culture medium supplemented with 8 µg/ml polybrene (Sigma–Aldrich, St. Louis, MO). Adherent cells were infected with similar conditions, although these were not subjected to spinfection. To generate EGFP-expressing cellines, 293T, HeLa, HT29, Raji, Jurkat, and THP-1 cells were infected with pSicoR-EGFP-T2A-Puromycin virus at low MOI (<0.1) to ensure single viral integrations. Individual EGFP-positive cells were single-cell sorted by using a Moflow cell sorter (Dakocytomation, Ft. Collins, CO) in 96 well plates, allowed to recover in the presence of puromycin selection medium and monitored for EGFP expression several weeks later. All clonal cellines uniformly expressed high levels of EGFP.

### Monitoring EGFP knockdown by shRNAs expressed from miR-30 backbone vectors

Stable EGFP-expressing cell lines were infected at low MOI (<0.2) with lentivirus expressing anti-EGFP shRNAs expressed from various polymerase II promoters described above or from the mouse polymerase pol-III U6 promoter as control. As additional controls, cells were infected with the same viruses lacking a miR-30-anti-EGFP unit. EGFP knockdown was monitored at various timepoints post-infection by flow cytometry on the FACSarray system (Becton Dickinson, CA). Percentage of EGFP knockdown was calculated by ((Geo-mean of uninfected cells minus Geo-mean of infected cells)/Geo-mean of uninfected cells)*100. Infected cells were discriminated from uninfected cells by means of mCherry-expression encoded on the lentiviral vector.

### Quantization of mature anti-EGFP siRNA expression

Cells stably expressing anti-EGFP shRNAs from the various polymerase II promoters were sorted by using a Moflow cell sorter (Dakocytomation, Ft. Collins, CO) based on mCherry marker expression. The cells were allowed to recover and were subsequently subjected to total RNA extraction by using the Trizol reagent according manufacturers recommendations (Invitrogen, Carlsbad, CA). Expression of the mature anti-EGFP strand was determined by real-time PCR essentially as described [28]. In short, 20 ng total RNA was reverse transcribed by using an EGFP-siRNA-specific stem-loop RT primer (5′-CTCAACTGGTGTCGTGGAGTCGGCAATTCAGTTGAGTGCAAGCT-3′) as described[28] in a MyCycler Thermal Cycler System at 16°C, 30 min at 42°C, 5 min at 85°C and then held at 4°C. Subsequently, Real-time PCR was performed as described[28] on an Applied Biosystems 7900HT Sequence Detection System (Applied Biosystems, Foster City, CA) in a 384-well plate at 95°C for 10 min, followed by 40 cycles of 95°C for 15 s and 58°C for 1 min. All reactions were run in triplicate. The EGFP forward oligo (5′-ACACTCCAGCTGGGGAACTTCAGGGTCAG-3′) and the EGFP-siRNA-specific probe 5′-56-FAM-TTCAGTTGAGTGCAAGCT-3IABLFQ-3′ were purchased from IDT DNA Technology (Coralville, IA, USA). The expression of the processed anti-EGFP siRNA was normalized to expression of the U6 snoRNA (Applied Biosystems, Foster City, CA) as determined by miRNA Taqman analysis according the manufacturer's instructions.
